# Computational Network Pharmacology, Molecular Docking, and Molecular Dynamics to Decipher Natural Compounds of *Alchornea laxiflora* for Liver Cancer Chemotherapy

**DOI:** 10.3390/ph18040508

**Published:** 2025-03-31

**Authors:** Nem Kumar Jain, Balakumar Chandrasekaran, Nasha’t Khazaleh, Hemant Kumar Jain, Moti Lal, Gaurav Joshi, Vibhu Jha

**Affiliations:** 1 School of Pharmacy, ITM University, Gwalior 474001, Madhya Pradesh, India; nemjain.pharma@itmuniversity.ac.in; 2 Faculty of Pharmacy, Philadelphia University, P.O. Box 1, Amman 19392, Jordan; nsk_92@outlook.com; 3 Department of General Medicine, Government Medical College, Datia 475661, Madhya Pradesh, India; 4 School of Sciences, ITM University, Gwalior 474001, Madhya Pradesh, India; motilal.bt@itmuniversity.ac.in; 5 Department of Pharmaceutical Science, Hemvati Nandan Bahuguna Garhwal University, Srinagar 246174, Uttarakhand, India; garvpharma29@gmail.com; 6 Institute of Cancer Therapeutics, School of Pharmacy and Medical Sciences, Faculty of Life Sciences, University of Bradford, Bradford BD7 1DP, UK

**Keywords:** *Alchornea laxiflora*, liver cancer, network pharmacology, molecular docking, molecular dynamics simulation

## Abstract

**Background:** *Alchornea laxiflora* (Benth.) Pax & K. Hoffm. (*A. laxiflora*) is utilized as a traditional herb for treating several diseases. **Objective**: Our study aims to identify the active phytochemical candidates from *A. laxiflora* and analyses to predict their anticancer activity mechanism by employing network pharmacology, molecular docking, and molecular dynamics (MD). **Methods**: The phytoconstituents of *A. laxiflora* were retrieved from the literature, and phytoconstituent-related targets implicated in hepatocellular carcinoma (HCC) were collected from respective databases. Computational methods were employed to recognize essential compounds, hub gene targets, and signaling pathways. **Results**: Our study has identified 12 potentially bioactive compounds, 150 potential anti-HCC targets, and 15 hub gene targets for *A. laxiflora*. Molecular docking results recognized the better binding energy values of below −5.6 kcal/mol. Further, MD simulations of the three of the top-scoring protein–ligand complexes (MAPK—3-acetylursolic acid, AKT1—quercetin, and AKT1—3-acetylursolic acid) allowed us to validate the docking results, evaluate the stability of the complexes, and associated conformational changes. **Conclusions**: Our research claims that phytoconstituents of *A. laxiflora* are crucial for treating liver cancer, and the recognized protein targets can serve as biomarkers, respectively.

## 1. Introduction

Liver cancer is a significant health challenge that is prevalent worldwide [1]. The most common form of liver cancer is hepatocellular carcinoma (HCC), which contributes 90% of all liver cancer cases. Unfortunately, the incidence of HCC is increasing globally, and there is predicted to be over a million new cases [2]. Other subtypes of liver cancer are intrahepatic cholangiocarcinoma (ICC) and mixed hepatocarcinoma. Different factors may contribute to the HCC, such as hepatitis B and C, non-alcoholic fatty liver disease, alcohol consumption, chronic liver disease, dietary factors, smoking, and environmental carcinogens like aflatoxin and aristolochic acid [3]. An infection with hepatitis B virus (HBV) is the primary reason for HCC, attributable to 50% of cases. Patients suffering from cirrhosis are still at high risk for developing HCC even after hepatitis C virus clearance [2]. Various genes have been implicated in HCC pathogenesis, including epigenetic regulation, oxidative stress, Wnt-beta-catenin signaling, Akt-mTOR signaling, and MAPK pathways [4].

Surgical resection and chemotherapy are two treatment options available for patients suffering from HCC. However, the delayed diagnosis of HCC leads to many patients progressing to advanced stages, which makes them resistant to chemotherapy [5]. Genetic and epigenetic factors influence the progress of HCC. Therefore, it is essential to study how abnormal gene expressions and molecular mechanisms contribute to the development of this cancer. Additionally, finding new complementary drugs that are effective, without side effects, and nontoxic to host cells is necessary for the chemotherapy. Recently, natural compounds have gained greater attention due to their potential anticancer properties, good accessibility, cost-effectiveness, and positive adjunctive effect with fewer or no side effects [6].

*Alchornea laxiflora* (Benth.) Pax & K. Hoffm. (*A. laxiflora*) is an understorey tree or shrub often found in deciduous woodlands or semi-deciduous tropical forests of eastern, central, and southern African countries. A wide distribution across Africa makes it an important part of the continent’s biodiversity. *A. laxiflora* is an integral part of the African traditional medicine system used by the natives for the remedy of hematological disorders, gastrointestinal disorders, infectious diseases, neurological disorders, and even cancer. Moreover, documented evidence suggests that *A. laxiflora* exhibited antibacterial, antiviral, antifungal, antidiabetic, anti-inflammatory, antioxidant, hepatoprotective, and anticancer properties. In Cameroon, *A. laxiflora* has traditionally been used in the treatment of jaundice and hepatitis [7]. In an in vitro investigation by Morah and Uduagwu, it was found that the petroleum ether and ethanolic extracts of leaves exhibited a greater percentage of radical scavenging activity than ascorbic acid at the dose of 200 μg/mL [8]. Additionally, there are multiple reports highlighting the anticancer activity of *A. laxiflora* [9]. Kuete et al. reported the potent anticancer activity of the methanolic extract of *A. laxiflora* stem and leaves against CCRF-CEM human T lymphoblast cell line with IC_50_ values of 49.21 and 43.67 μg/mL, respectively [10]. Furthermore, an in vivo study was conducted using rats to rationalize the hepatoprotective effects in folklore medicine. The results showed that the ethyl acetate extract of *A. laxiflora* (100 mg/kg body weight) significantly counteracted the CCl_4_-induced liver damage by lowering the levels of elevated marker enzymes, namely, ALT, AST, ALP, and LDH levels in the blood [11]. The inclusion of *A. laxiflora* in the diet or therapeutic regimen prevents oxidative stress and chronic hepatitis, both of which are major risk factors in the pathogenesis of HCC. Still, there is no supporting pharmacological evidence for the anti-HCC activity of *A. laxiflora*. Hence, we have employed network pharmacology, molecular docking, and molecular dynamics simulations to explore potential anti-HCC phytocompounds of *A. laxiflora*.

Network pharmacology is an innovative and promising approach to establishing correlations between drugs and diseases at the system and biological network levels. It helps gain a more comprehensive understanding of the relationship between various drugs and diseases [12]. A large body of evidence suggests the importance of network pharmacology in understanding multi-component and multi-pathway mechanisms of traditional drugs in the systems of Chinese medicine and Indian Ayurvedic medicine [13]. Similarly, molecular docking aids in identifying molecular interactions, predicting receptor–ligand complex structures, and verifying functional components in drug structures [14]. On the other hand, molecular dynamics offers knowledge regarding the drug molecules (natural or synthetic) binding to their corresponding protein targets to estimate the kinetics and free energy of binding. In this research, we, therefore, employed various computational methods of network pharmacology, molecular docking, and molecular dynamics simulations to envisage bioactive compounds, key molecular targets, and pathways to identify and analyze the possible anticancer properties of *A. laxiflora*.

## 2. Results

### 2.1. Screened Bioactives of A. laxiflora

Active components of *A. laxiflora* were collected from our previous study [15]. Finally, out of 132 compounds, 14 compounds were screened for further investigation based on OB ≥ 30% and DL ≥ 0.18 screening criteria. All of the selected compounds complied with Lipinski’s rule of five with no more than one violation. General information on screened bioactives is collected in Table 1.

### 2.2. Potential Targets of A. laxiflora in the Treatment of Liver Cancer

**Figure 1:**
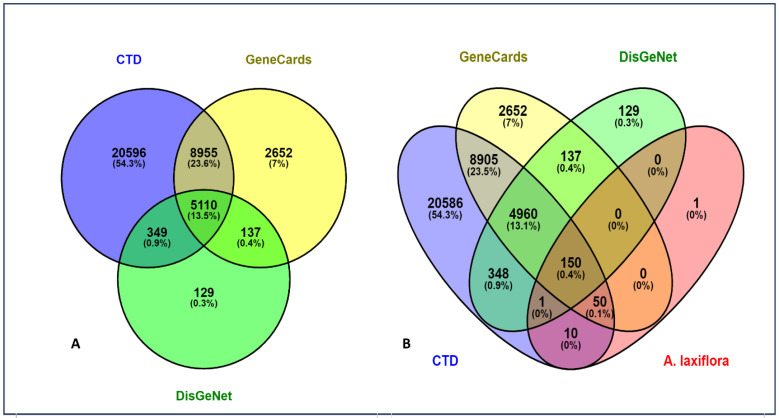
(**A**) Targets of HCC and (**B**) common targets between *A. laxiflora* and HCC.

The potential bioactive compound targets were predicted using the BindingDB and Swiss Target Prediction databases with “*Homo sapiens*” (limitation). After merging and de-duplication, 213 potential targets were retrieved from both databases with a probability/similarity score of >70%. Among the screened bioactive compounds, 12 components yielded potential targets, and two catered to none. Byzantionoside B and phaeophorbide A have no predicted targets. HCC-related proteins were retrieved from the databases of CTD, GeneCards, and DisGeNet resulting in 35,011, 16,854, and 5725 predicted targets. With the help of Venny 2.1.0, false positives were identified and eliminated to yield a set of 5110 HCC targets exhibiting more accuracy and reliability (Figure 1A). Further, the targets were intersected with the acquired HCC targets to identify potential therapeutic targets for *A. laxiflora*. Finally, 150 *A. laxiflora* targets were obtained (Figure 1B).

### 2.3. BA-TAR Network Construction

**Figure 2:**
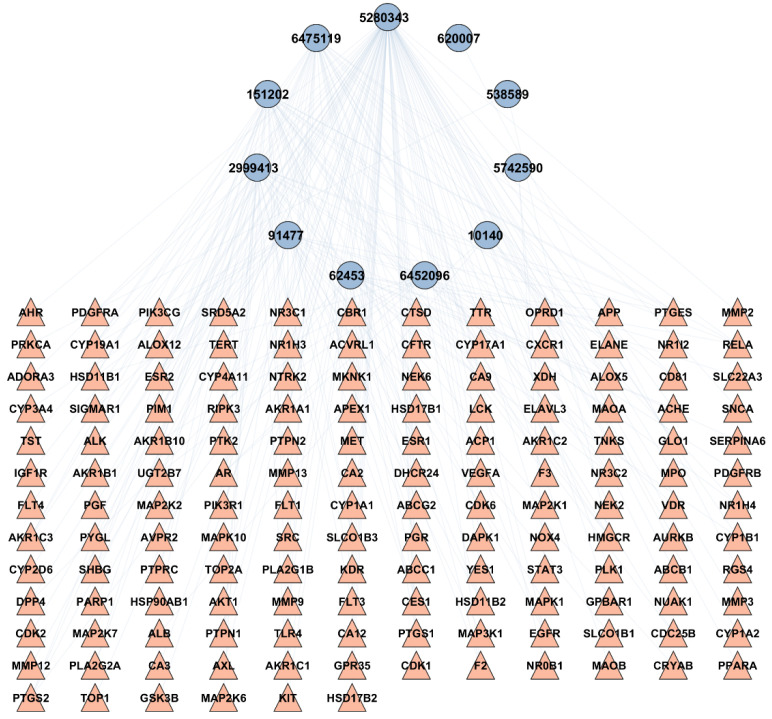
BA-TAR network with 161 nodes and 239 edges linking screened bioactive compounds with HCC targets. Blue-colored nodes indicate 11 bioactives and pink triangles depict 150 HCC targets.

To examine the correlation between the bioactives of *A. laxiflora* and the overlapping targets of HCC and *A. laxiflora* bioactives, a BA-TAR network was created using Cytoscape v3.9.1 software. A total of 150 potential targets and 12 bioactive compounds were added to Cytoscape v3.9.1 to construct a bioactive-target network. The network analyzer was utilized to determine the number of nodes and edges in the constructed network. A degree analysis was performed on the 12 bioactives in the BA-TAR network and suggested that quercetin, 3-acetylursolic acid, and 3-acetyloleanolic acid were the bioactive compounds of *A. laxiflora*, associated to 94, 35, and 34 genes, respectively (Table 2). These three bioactive compounds were selected further for the molecular docking study. Figure 2 demonstrates the resulting network consisting of 161 nodes and 239 edges with an average of 2.97 neighbors per node. Each bioactive compound interacted with various gene targets, indicating the multi-target synergistic effect of *A. laxiflora*.

### 2.4. PPI Network Analysis

**Figure 3:**
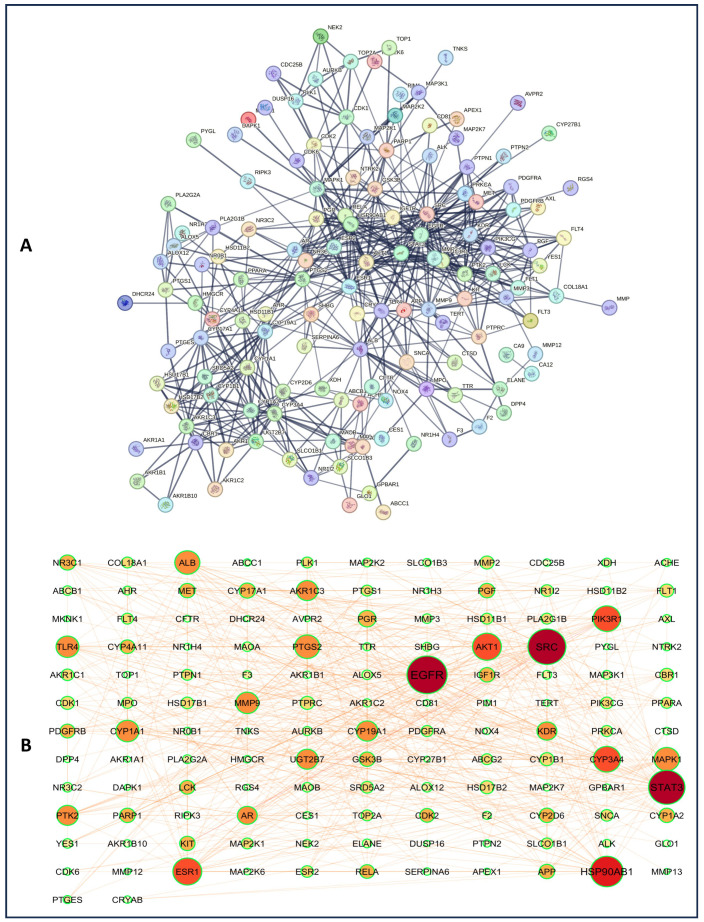
PPI network of potential anti-HCC targets. (**A**) STRING database PPI network (confidence score ≥ 0.70) and (**B**) Cytoscape v3.9.1-mapped PPI network. Nodes depicting the target and edges represent the interaction between protein targets. The darker and larger nodes correspond to the higher degree and greater therapeutic importance.

**Figure 4:**
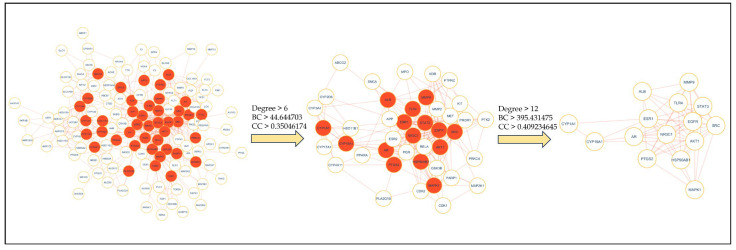
Hub gene screening.

Intersecting targets (150) of HCC and *A. laxiflora* were mapped to the STRING database with a limitation to “*Homo sapiens*” to obtain a PPI network that consists of 150 nodes and 536 edges. Figure 3A presents that the resulting PPI network has an average local clustering coefficient of 0.52 and an average node degree of 7.15. The STRING results were further imported to Cytoscape v3.9.1 software to visualize the PPI network, as shown in Figure 3B. The visualization results established that the PPI network comprised 134 nodes and 535 edges after removing 16 disconnected nodes. Further, to extract the hub gene targets, the first core targets were screened from the entire dataset based on the cutoff criteria, [the genes with a median value greater than the degree value (6), BC value (44.644703), and CC value (0.35046174)] to obtain 42 core targets. Later, 15 hub genes, including EGFR, SRC, STAT3, HSP90AB1, AKT1, ESR1, PTGS2, MAPK1, ALB, TLR4, MMP9, CYP1A1, CYP19A1, AR, and NR3C1, were identified with the screening criteria of the genes with a median value greater than the degree value (12), betweenness centrality value (395.431475), and closeness centrality value (0.409234645) (Figure 4). These targets were top nodal targets, showing strong association with potential targets and believed to have a critical role in treating liver cancer/HCC. Table 3 presents the general description of the PPI network hub genes.

### 2.5. GO and KEGG Enrichment Analysis

**Figure 5:**
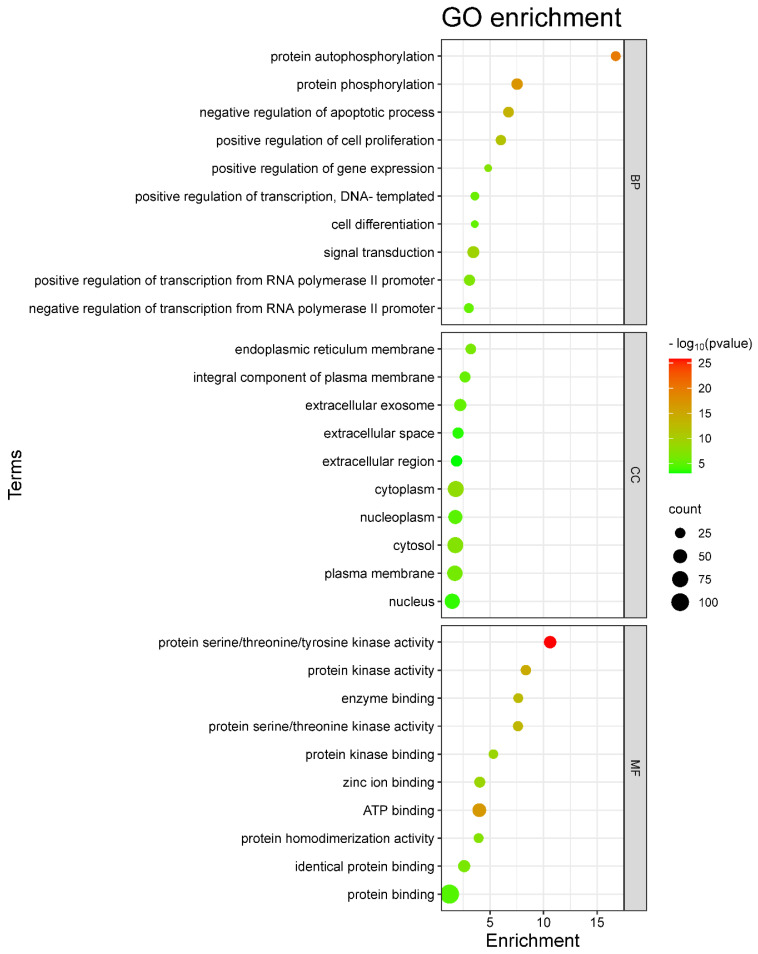
GO annotation chart.

The 150 overlapping targets of HCC and *A. laxiflora* were scrutinized with the DAVID 2021 functional annotation tool to investigate the mechanism behind the therapeutic effects of *A. laxiflora* on HCC at the molecular level. Examination revealed substantially enriched 112 BP terms, 23 CC terms, and 59 MF terms based on *p* < 0.01 and FDR < 0.01 values. The top 10 significant BP, CC, and MF terms were identified and drawn as bubble plots (Figure 5). The size of the bubbles in the chart corresponds to the number of target genes. At the same time, the color indicates the proportion of target genes among all annotated genes. The top three enrichment terms for BPs were signal transduction (GO:0007165), protein phosphorylation (GO:0006468), and positive transcriptional regulation involving RNA polymerase II promoter (GO:0045944). On the other hand, for CCs, the top three enrichment terms were cytosol (GO:0005829), cytoplasm (GO:0005737), and plasma membrane (GO:0005886). Finally, the top three MF enrichment terms are protein binding (GO:0005515), ATP binding (GO:0005524), and protein serine/threonine/tyrosine kinase activity (GO:0004712).

**Figure 6:**
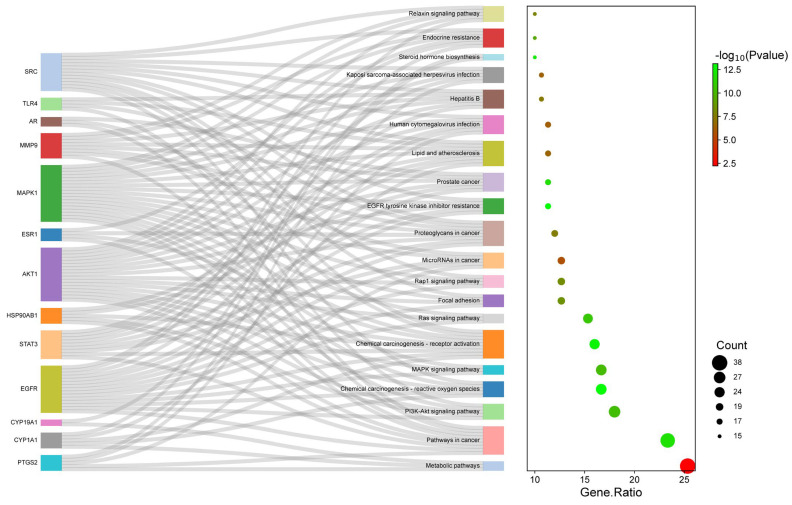
KEGG pathway analysis.

The KEGG pathway analysis was accomplished on 150 intersecting targets to deduce 98 enriched pathways at *p* < 0.01 and FDR < 0.01 and screen the top 20 pathways (Figure 6). Notably, most of the key genes were enriched in the screened pathways, including hsa01100 metabolic pathways (38), hsa05200 pathways in cancer (35), hsa04151 PI3K-Akt signaling pathway (27), hsa05208 chemical carcinogenesis-reactive oxygen species (25), and hsa04010 MAPK signaling (25) as the top pathways. Table 4 depicts the details of the top 10 highly enriched KEGG pathways.

The finding suggests that *A. laxiflora* can treat HCC by regulating the hsa04151 PI3K-Akt signaling pathway, hsa05208 chemical carcinogenesis-reactive oxygen species pathway, and hsa04010 MAPK signaling pathway. A couple of other pathways, such as hsa04014 Ras signaling and hsa04015 Rap1 signaling, also support the possible therapeutic applications of *A. laxiflora* in the treatment of liver cancer.

### 2.6. BA-TAR-PATH Network Construction

**Figure 7:**
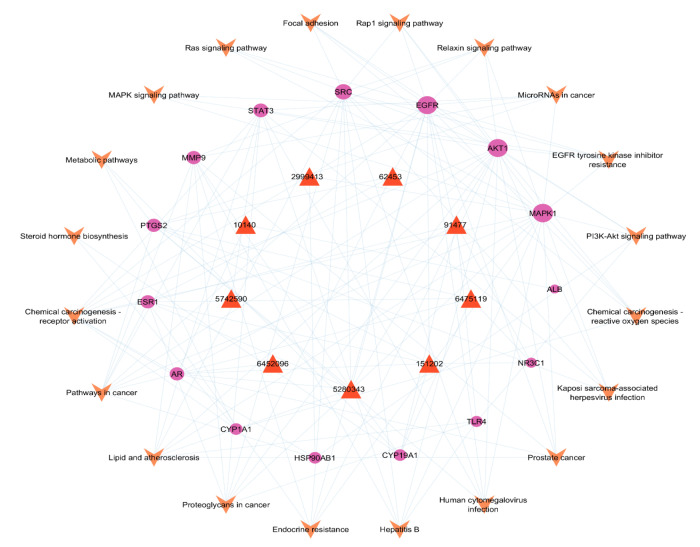
BA-TAR-PATH network.

A visual network called BA-TAR-PATH was created using Cytoscape v3.9.1 to analyze the connection between bioactives, potential therapeutic hub targets, and critical KEGG analysis pathways. Figure 7 shows the 44 nodes (9 bioactive compounds, 15 targets, and 20 pathways) and 141 edges in the network. The network revealed that most targets were influenced by at least one bioactive molecule, and all the hub genes were possibly involved in HCC-related targeted pathways. According to the degree analysis, MAPK1 (19), AKT1 (18), EGFR (18), SRC (13), STAT3 (10), MMP9 (9), PTGS2 (9), and ESR1 (9) showed the highest degree of association with multiple bioactives and pathways. This advocates the potential role in the mechanism of *A. laxiflora* bioactives against HCC. As a result, these eight proteins were considered potential targets for molecular docking.

### 2.7. Clusters Network Analysis

**Figure 8:**
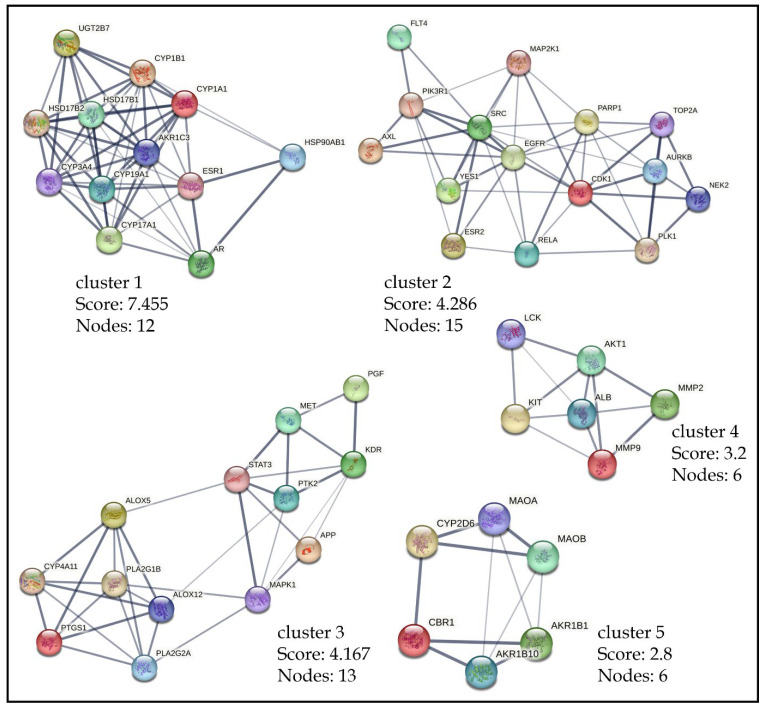
Cluster analysis of the intersecting protein targets within the PPI network.

To validate KEGG enrichment analysis and PPI network analysis, a cluster network analysis was executed on the intersected targets using the MCODE plugin (Cytoscape v3.9.1). The top four targets reported in Section 2.6 were found to be clustered in the five obtained modules, as shown in Figure 8. MAPK1 was clustered in cluster 3, while cluster 4 had AKT1 as the core target. Similarly, EGFR and SRC were the core nodes in the cluster 2. The results corroborate the findings of the BA-TAR-PATH network, suggesting these four targets as the core targets.

### 2.8. Molecular Docking

**Figure 9:**
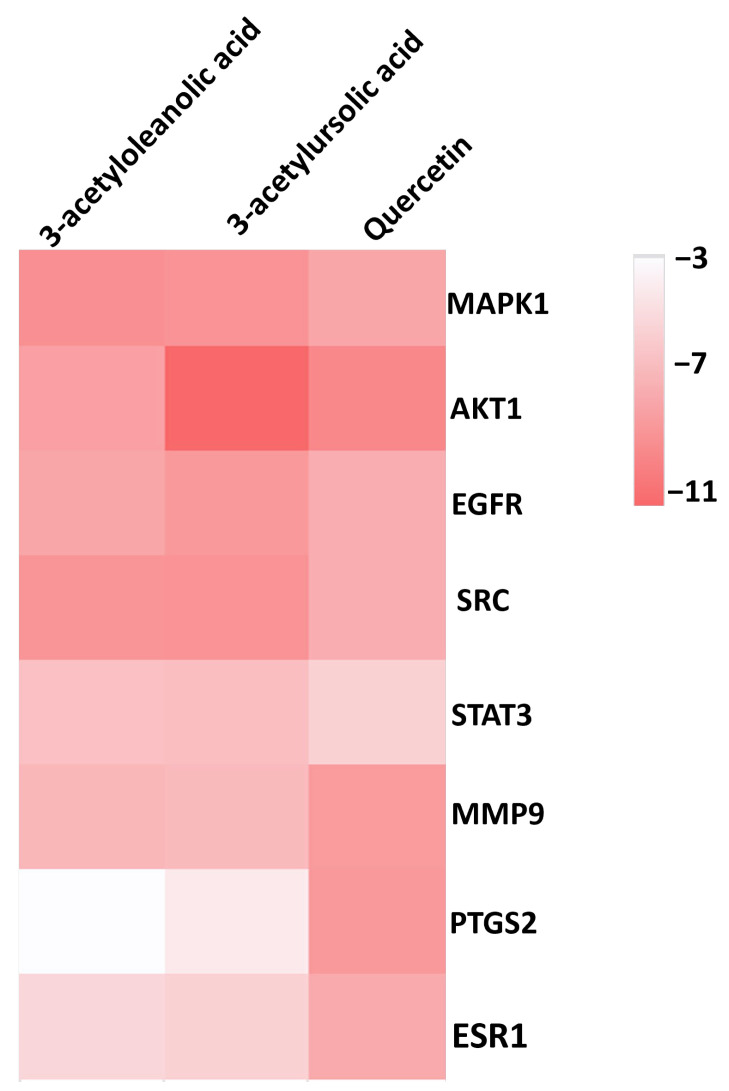
Clustering heatmap of binding energies (kcal/mol) from molecular docking. The darker the color, the higher the free energy for the phytocompounds to bind to the hub targets.

Based on the top ten hub gene targets and the proteins implicated in the signaling pathways, eight protein targets (MAPK1, AKT1, EGFR, SRC, STAT3, MMP9, PTGS2, and ESR1) were selected for the docking simulation against three ligands (Quercetin, 3-acetylursolic acid, and 3-acetyloleanolic acid) from *A. laxiflora*. The binding energy values ranged from −3.6 to −11.6 kcal/mol for the docked complexes. Figure 9 illustrates the clustering heatmap of molecular docking binding energies. In general, the binding energy determines the stability of target–ligand complexes. If the binding energy is lower, it indicates the better stability of the formed target-ligand complexes through stronger molecular interactions between them. A binding energy of less than −5 kcal/mol displays a higher binding affinity of the ligand towards the target protein, which leads to better pharmacological properties [16]. The results of docking of three ligands against eight proteins showed a binding energy of less than −5.6 kcal/mol for all the studied complexes. Specifically, MAPK1-3-acetylursolic acid, AKT1-quercetin, and AKT1-3-acetylursolic acid complexes exhibited the best binding energy values of −9.2, −9.8, and −11.5 kcal/mol (Table 5), respectively.

### 2.9. Molecular Dynamics Simulations

MD simulations were carried out to evaluate dynamic stability and conformational changes associated with the selected protein–ligand complexes based on the best docking scores. Three complexes (MAPK—3-acetylursolic acid, AKT1—quercetin, and AKT1—3-acetylursolic acid) were selected for a 100 ns MD simulation study, starting from the docking poses of ligands. As a measure of ligand mobility, the root-mean-square deviations (RMSD) of the ligand-heavy atoms were calculated. On the other hand, the RMSDs of the protein α-carbons were calculated as a measure of protein mobility. Further, we have calculated RMSFs (root-mean-square fluctuations) of protein and ligand to monitor the fluctuation at the residue level.

**Figure 10:**
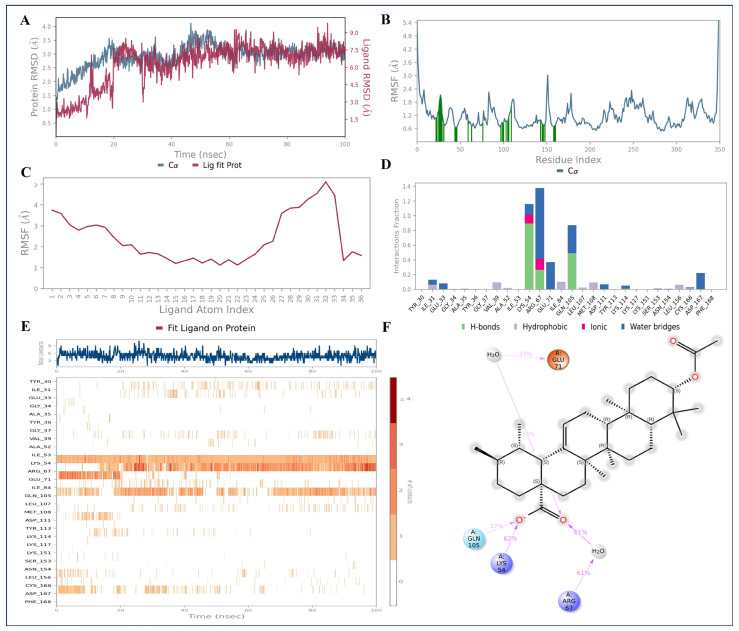
The 100 ns MD simulation analysis of MAPK—3-acetylursolic acid complex: (**A**) protein—ligand RMSD; (**B**) protein RMSF; (**C**) ligand RMSF; and (**D**–**F**) protein–ligand interaction diagrams.

Starting with the MAPK—3-acetylursolic acid complex, the RMSD plot for protein revealed that for the initial 20 ns, protein showed a slight fluctuation. After that, it remained stable throughout simulation time possessing RMSD values within 3.0 Å (Figure 10A). Adopting a similar conformational change pattern as of the protein, 3-acetylursolic acid demonstrated an initial fluctuation till ∼20 ns, followed by acquiring stable RMSD till 100 ns simulation, showing RMSD values within 7–7.5 Å (Figure 10A), which further confirmed that 3-acetylursolic acid stays in the MAPK binding site during the simulation. In addition, the RMSF analysis indicated that the changes or areas of protein fluctuations majorly during simulation. RMSF analysis also revealed that fluctuations were observed at the 159^th^ amino acid residue (Thr159) up to 3 Å (Figure 10B). The ligand RMSF showed the perturbation in the carboxylic acid unit of the 3-acetylursolic acid (Figure 10C). The protein–ligand interaction histogram of MAPK—3-acetylursolic acid complex showed that Lys54, Arg67, and Gln105 formed hydrogen bonds with the ligand carboxylic group, for 100%, 48%, and 50% of the simulation time (Figure 10D–F).

**Figure 11:**
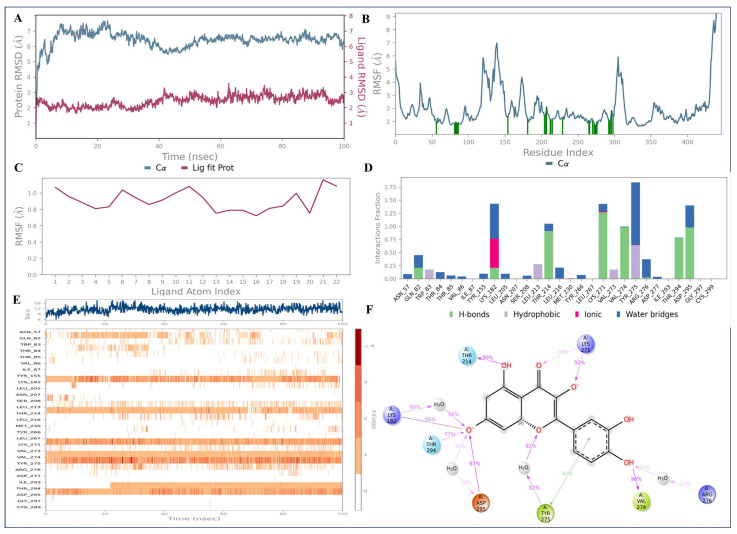
The 100 ns MD simulation analysis of AKT1—quercetin complex: (**A**) protein–ligand RMSD; (**B**) protein RMSF; (**C**) ligand RMSF; and (**D**–**F**) protein–ligand interaction diagrams.

The RMSD of the AKT1—quercetin complex demonstrated some fluctuations with the protein atoms till 40 ns of the trajectory; however, it remained stable afterwards, demonstrating RMSD values between 4 and 6 Å (Figure 11A). Interestingly, the ligand quercetin remained greatly stable in the AKT1 binding site throughout the 100 ns simulation time, possessing RMSD values within 2–3 Å range (Figure 11A). Protein RMSF indicated the fluctuations were found with Met121, Ser140, and Gly306 (Figure 11B). No major fluctuations were noted with the ligand RMSF plot, which agrees with the protein–ligand RMSD plots (Figure 11C). The protein–ligand contact histogram revealed that Lys182 (66%), Thr214 (90%), Lys271 (92%), Thr294 (77%), Asp295 (97%), and Val274 (98%) were involved in H-bonding interactions with the bicyclic system of the quercetin, engaging its hydroxy and carbonyl groups. The aromatic ring of Tyr275 formed π-π stacking interactions with the aromatic ring (1–2 dihydroxyphenyl) of the quercetin (Figure 11D–F).

**Figure 12:**
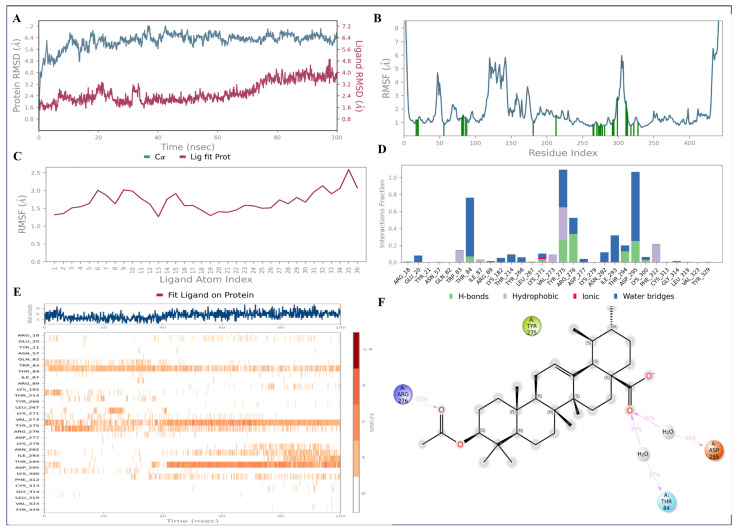
The 100 ns MD simulation analysis of AKT1—3-acetylursolic acid complex: (**A**) protein–ligand RMSD; (**B**) protein RMSF; (**C**) ligand RMSF; and (**D**–**F**) protein–ligand interaction diagrams.

MD analysis of the AKT1—ursolic acid complex demonstrated notable stability over the course of 100 ns simulation, with no major fluctuations. The protein atoms displayed RMSD values within the range of 4–6 Å, while the ligand atoms demonstrated RMSD values in the range of 2–4 Å during the simulation time, confirming the conformational stability (Figure 12A). The RMSF indicated that fluctuations were found between 122nd and 142nd amino acid residues and between 306^th^ and 308^th^ residues (Figure 12B). The ligand RMSF analysis showed the perturbation occurs only at the terminal methyl ester unit of the ursolic acid. The protein–ligand contact histogram revealed that Thr84 (37%), Tyr275 (30%), Arg276 (33%), and Asp295 (35%) are involved in H-bonding through the carboxylic acid group of the ligand (Figure 12D–F).

MD simulation analysis of all the three best complexes confirmed the stability and robustness of the structural models, further validating the overall studies carried out in this research.

### 2.10. Anti-HCC Hub Gene Expression and Prognosis of Liver Cancer Patients

**Figure 13:**
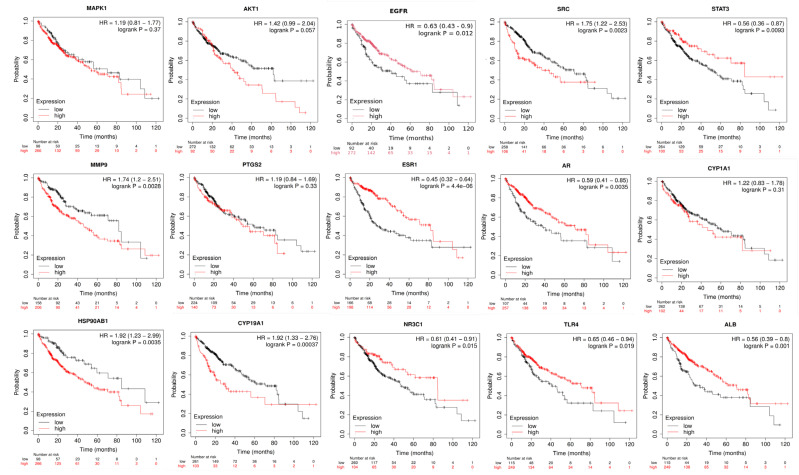
KM plotter analysis of the relationship between hub target gene expression and HCC patient survival.

The study also aimed to determine if the hub targets of *A. laxiflora*, which act on HCC, could be used as molecular markers to predict HCC. To achieve this, we have used the Kaplan–Meier mapping (KM) to determine the association between the expression levels of fifteen hub target genes and the survival of liver cancer/HCC patients (Figure 13). According to the investigation, patients with high expression of EGFR, STAT3, ESR1, AR, NR3C1, TLR4, and ALB target genes showed better overall survival (OS). Patients with low expression of SRC, MMP9, HSP901AB1, and CYP19A1 genes had significantly higher OS than those with high expression of these genes (*p* < 0.001). Moreover, patients with high expression of the genes STAT3, ESR1, and AR had significantly higher survival rates than those with low expression of these genes (*p* < 0.01). On the other hand, PTGS2, MAPK1, AKT1, and CYP1A1 gene expression did not show any significant effect on the OS of the patients.

## 3. Discussion

Liver cancer (or) HCC is the most common type of liver cancer in the Asia-Pacific region, and it accounts for the majority of liver cancer-related deaths [17]. Despite extensive research on chemotherapy and immunotherapy, patients still suffer from severe toxicity and side effects [18]. However, medicinal plants have the potential to provide critical bioactive compounds that can target multiple oncogenic pathways exhibiting minimal or no side effects. African traditional medicine system has used *A. laxiflora* as an antioxidant and hepatoprotective herb for centuries. However, there is still limited knowledge about the potential anti-HCC properties and mechanism of action of *A. laxiflora* to treat liver cancer. The current study presents a theoretical framework for future research on early screening of bioactive compounds obtained from *A. laxiflora* and deducing the pathways/mechanisms involved in the treatment of liver cancer.

Our study involved computational approaches of network pharmacology, molecular docking, and molecular dynamics to gain insight into molecular interactions and the possibility of liver cancer chemotherapy. Initially, bioactive compounds of *A. laxiflora* were retrieved from our earlier published study [15]. To investigate their therapeutic applications, the collected compounds were screened for their bioavailability and drug-like scores, and their possible targets were collected from public repositories. A total of 12 bioactive compounds and 213 targets were retrieved. The disease-related data on HCC were also collected from different databases, resulting in a 5110 target database. Subsequently, the 150 overlapping gene targets obtained from comparing the plant-related targets with the HCC-related targets were taken to a network pharmacology method to analyze the multi-target effect of potential bioactive compounds against HCC. According to BA-TAR pathway network analysis, all the phytocompounds demonstrated good potential to bind with target proteins associated with HCC.

We have identified and screened three active compounds (quercetin, 3-acetylursolic acid, and 3-acetyloleanolic acid) as suitable compounds conferring anti-HCC activity since these compounds were reported for antioxidant and antitumor activities. In an in vitro and in vivo investigation by Wu et al., quercetin inhibited HCC growth by regulating cell apoptosis, autophagy, migration, and invasion mediated via JAK2/STAT3 signaling pathway [19]. Quercetin has been shown to have anti-HCC activity associated with apoptosis induction via activating the MAPK pathway and inhibiting AKT/mTOR pathway [20]. The compound 3-acetyloleanolic acid is reported to exert anticancer activity in different cancer types by modulating angiogenesis, lymphangiogenesis, apoptosis, and metastasis. Hwang-Bo et al. documented the inhibitory potential of 3-acetyloleanolic acid on tumor-induced angiogenesis and lymph-angiogenesis via suppression of angiopoietin-1/Tie-2 signaling and downstream signaling factors such as AKT, FAK, and ERK1/2 in CT-26 allograft colon carcinoma animal model [21]. This derivative of oleanolic acid is also reported to exert therapeutic benefit against hyperlipidemia in non-alcoholic fatty liver disease in rats through AMP-activated protein kinase (AMPK)- related pathways [22]. The compound 3-acetylursolic acid is a 3-acetylated ursolic acid derivative, a potent anticancer natural dietary compound. AlQathama et al., in an in vitro investigation, compared the anti-proliferative and anti-migratory activity of ursolic acid and 3-acetylursolic acid, alone or in combination with quercetin, in melanoma A375 cell lines [23]. 3-acetylursolic acid was found to exert equipotent anti-proliferative activity like ursolic acid.

The analysis of the PPI network revealed that *A. laxiflora* phytoconstituents may target 15 hub genes to treat HCC. These potential targets, including EGFR, SRC, STAT3, HSP90AB1, AKT1, ESR1, PTGS2, MAPK1, ALB, TLR4, MMP9, CYP1A1, CYP19A1, AR, and NR3C1, were involved in the molecular mechanism of HCC pathogenesis. The BA-TAR-PATH network analysis identified EGFR, AKT1, MAPK1, and SRC as the most significant genes in the pathogenesis of HCC. The EGFR pathway is critical because it can initiate several signaling cascades, such as NF-kB, the Ras/Raf/MEK/MAPK cascade, and the ERK-PI3K-Akt pathway [24]. These pathways are essential in regulating the inflammatory microenvironment, tumor proliferation, epithelial–mesenchymal transition, differentiation, and angiogenesis in HCC.

EGFR inhibition is the primary mechanism of anticancer activity of the drug sorafenib [25]. AKT1 overexpression is associated with the development of HCC [26]. It triggers the phosphorylation of mTORC2, which is a crucial factor in HCC progression in both mice and humans. The PI3K/AKT/mTOR pathway was reported to facilitate cancer cell growth and metastasis [27]. Therefore, various inhibitors are currently under clinical trials targeting this pathway in liver cancer patients [28]. In HCC cells, MAPK1 activation is reported in the anti-apoptotic functions and drug resistance. MAPK/ERK signaling pathway is frequently overactivated in more than 50% of cases of early and advanced stages of HCC [29,30]. A variety of modulators have been implicated in the activation of MAPK/ERK signaling, such as RAS-GAP, RAS-GEF, growth factors (EGF, FGF, HGF, IGF), and hepatitis virus (HBV and HCV) [29]. Therefore, MAPK/ERK signaling-based targeted therapies are emerging for liver cancer chemotherapy. Similarly, SRC is an oncogene whose overexpression or elevated activity is associated with HCC tumor progression and metastasis. Zhao et al. carried out an immunohistochemical analysis on 52 northern Chinese patients with HCC to determine the expression level of total-Src and phosphorylated p-Y416Src [31]. The results revealed a significantly elevated total and phosphorylated Src expression in HCC tissues compared with the non-HCC tissues. Owing to the crucial role of Src signaling in the pathogenic events of HCC, including proliferation, invasion, angiogenesis, and drug resistance, the United States Food and Drug Administration (FDA) has approved saracatinib, an Src inhibitor, as the first targeted therapy for the treatment of HCC [32]. STAT3 inhibition is a potential strategy for HCC treatment, inducing immunogenic cell death (ICD) by blocking glycolysis. Li et al. reported that targeting STAT3 triggers ICD in HCC cells, stimulates anti-HCC immune responses, and enhances immune memory in vivo [33]. Another hub gene, HSP90AB1, has been identified as a key immune-related gene in HCC prognosis. A recent study, incorporating both in vitro and in vivo experiments, reported that the CDK1-SRC interaction-driven transcriptional activation of HSP90AB1 enhances antitumor immunity in HCC [34]. ESR1 has been identified as a crucial hub gene and prognostic marker in HCC. Its overexpression has been associated with the suppression of HCC proliferation and invasion. A recent study revealed that dihydrotanshinone I, a compound derived from *Salvia miltiorrhiza*, targets ESR1, leading to its overexpression, induction of DNA double-strand breaks, and inhibition of HCC proliferation [35]. PTGS2, or Cyclooxygenase 2 (COX-2), is a commonly overexpressed prognostic marker in HCC. A recent study found that inhibiting mitochondrial COX-2 improves chemosensitivity in HCC by modulating mitochondrial dynamics through dynamin-related protein 1 (Drp1) [36]. Elevated MMP9 activity in HCC drives the proteolytic cleavage of MHC class I chain-related protein A (MICA), leading to the release of soluble MICA and enabling tumor immune evasion [37]. These molecular targets are believed to play a crucial role in carcinogenesis and drug resistance. Targeting these proteins and associated pathways with new molecular inhibitors could be a promising therapeutic approach to effectively treat liver cancer. In alignment with that, the phytoconstituents in *A. laxiflora* can act on those potential protein targets.

The GO enrichment analysis of 150 intersecting targets demonstrated that *A. laxiflora* might display an anti-HCC effect through binding to targets involved in signal transduction, protein phosphorylation, negative regulation of the apoptotic process, cell differentiation, and positive regulation of cell proliferation. Kinase-dependent protein phosphorylation is the key regulatory mechanism in the HCC development and progression [38]. Similarly, those targets are also present in the other cellular components. These HCC targets are implicated in multiple molecular functions, such as ATP binding, protein binding, and protein serine/threonine/tyrosine kinase activity.

Our study demonstrated multiple signaling pathways implicated in the anti-HCC mechanism of *A. laxiflora* phytochemicals through KEGG enrichment analysis. The hub gene targets were significantly enriched in the PI3K-Akt, MAPK, chemical carcinogenesis-reactive oxygen species, and Ras and Rap1 signaling pathways, indicating the significance of these pathways in HCC. By targeting these pathways and associated pathological processes, *A. laxiflora* can have a potential role as an anti-HCC herb. PI3K-Akt signaling has been linked to multiple pathological events in the HCC such as tumorigenesis, angiogenesis, proliferation, endothelial–mesenchymal transition, invasion, and metastasis. Moreover, activation of PI3K-Akt signaling mediates radiotherapy and chemotherapy resistance [39]. Previous studies reported that the suppression of PI3K-Akt signaling can result in the inhibition of cell proliferation, the induction of cell apoptosis, and autophagy [40]. MAPK signaling pathway is closely related to the oncogenesis, tumor progression, and drug resistance in a variety of cancers including HCC [41]. Huang et al. described that HIGD2A silencing impaired HCC growth via attenuating activated MAPK/ERK pathways [42]. In a separate study, butorphanol was found to have anti-angiogenic and anti-metastatic effects in mice by suppressing MAPK signaling [43]. The Ras signaling pathway is another dominant signaling network in HCC pathogenesis that is responsible for promoting cell proliferation and survival. Ras activation regulates the genes involved in the proliferation and survival of HCC cells. Newell et al. reported hyperactivation of the Ras pathway in 10.3% of cases of HCC [44]. Sorafenib and rapamycin synergistically blocked Ras signaling and displayed anticancer activity. Several network pharmacology studies also suggested that targeting these pathways can effectively treat HCC [45,46]. Therefore, our study has found that the active phytoconstituents of *A. laxiflora* and its associated protein targets can potently and synergistically treat liver cancer/HCC. The molecular docking study confirmed good binding interactions between targets and the phytoconstituents of *A. laxiflora*. The important phytoconstituents quercetin, 3-acetylursolic acid, and 3-acetyloleanolic acid displayed excellent binding energy against the selected targets, and the results indicated that those phytoconstituents can form strong complexes, which could be crucial in liver cancer therapy. To further validate the molecular docking, we have carried out a 100 ns MD simulation of the best three complexes (MAPK—3-acetylursolic acid, AKT1—quercetin, and AKT1—3-acetylursolic acid). MD analysis confirmed the dynamic stability of these complexes and highlighted the conformational changes and differences concerning each other, paving the way for further optimization.

The Kaplan–Meier plotter tool allows for a comprehensive assessment of the impact of over 54,000 genes on the prognosis of 21 different cancers. We analyzed the survival curves of 15 core target genes of *A. laxiflora* in HCC using the Kaplan–Meier plotter tool. The results unveiled that the patients with high levels of SRC gene expression had lower survival rates than those with low levels (*p* < 0.001). Conversely, patients with high levels of EGFR gene expression had higher survival rates than those with low levels (*p* < 0.001). López-Luque et al. described that the downregulation of EGFR could facilitate the TGF-β induced epithelial–amoeboid transition and pro-migratory and invasive functions in HCC [47]. Therefore, it can be concluded that EGFR and SRC genes may serve as reliable indicators of survival prognosis in HCC patients.

Our research is focused on the identification and analysis of the mechanism of action of *A. laxiflora* in treating liver cancer, using the combined computational efforts involving network pharmacology, molecular docking, and molecular dynamics. In our previous study, we aimed to rationalize the ethnomedicinal antidepressant activity of *A. laxiflora* using a similar methodology, identifying SRC, EGFR, PIK3R1, AKT1, and MAPK1 as core targets. 3-Acetyloleanolic acid and 3-acetylursolic acid emerged as the most active compounds with antidepressant potential, and molecular docking analysis was performed to validate these findings [48]. In this study, we explored another ethnomedicinal use of this understudied plant, employing a similar approach but incorporating molecular dynamics (MD) simulation for further validation. Compared to our previous study, molecular docking results were additionally verified through MD simulation, revealing that key bioactive compounds of *A. laxiflora*, quercetin and 3-acetylursolic acid, form stable complexes with AKT1, a key target in HCC pathogenesis.

The present study has yielded important insights into the possible anticancer mechanisms of *A. laxiflora*. Our results also demonstrated that *A. laxiflora* has great potential in interacting with key protein targets and exerting the possible anticancer effects against liver cancer/HCC.

## 4. Materials and Methods

**Figure 14:**
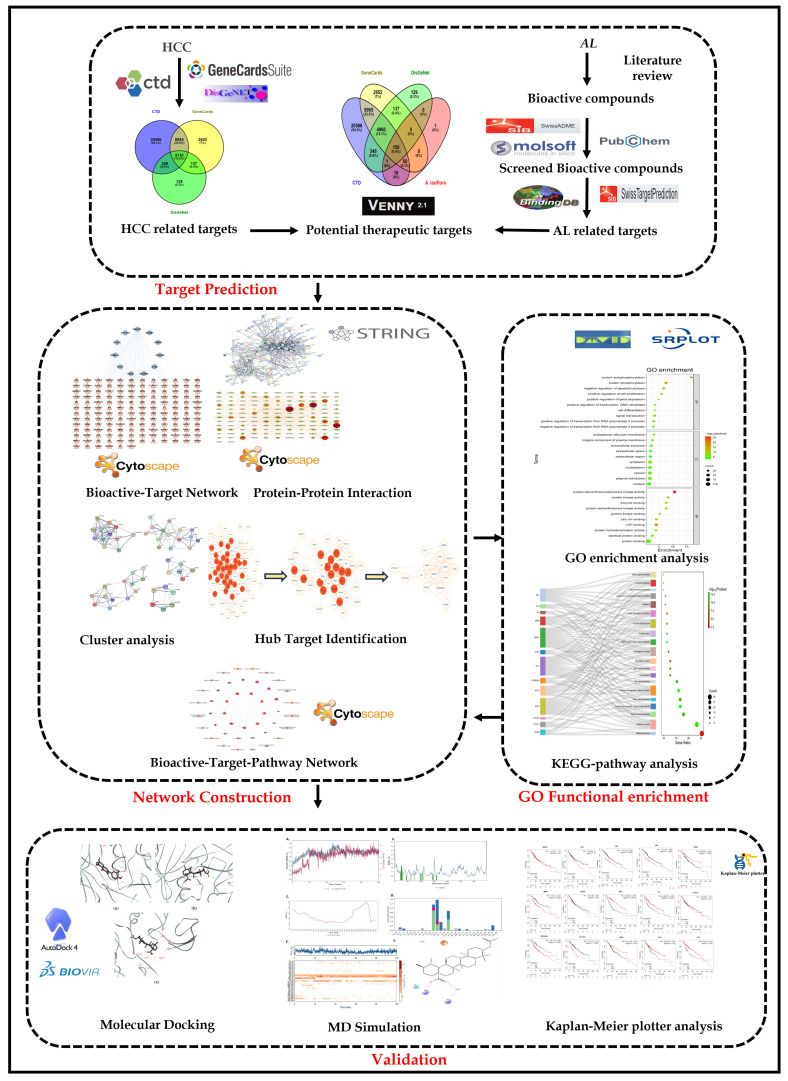
The methodological layout of the present study.

A detailed methodological layout of the presented study is given in Figure 14.

### 4.1. Collection of A. laxiflora Bioactive Compounds

From our earlier published studies, we have retrieved the details of the bioactive compounds of *A. laxiflora* [15] and computed their pharmacokinetic ADME properties using SwissADME [http://www.swissadme.ch/index.php (accessed on 5 August 2023)] and Molsoft [https://molsoft.com/mprop/ (accessed on 7 August 2023)]. To accomplish this, the Simplified Molecular Input Line Entry System (SMILES) notations of the bioactive compounds have been entered, followed by a screening in the PubChem database [https://pubchem.ncbi.nlm.nih.gov/ (accessed on 10 February 2023)] [49,50,51]. In this research, phytocompounds exhibiting scores of oral bioavailability (OB) ≥ 30% and drug likeliness (DL) ≥ 0.18 were selected for further investigations. The computation of ADME properties was also conducted based on Lipinski’s rule of five, and any bioactive ligands having more than three violations were not considered for further studies [52].

### 4.2. Screened Bioactives’Target Prediction

By using the SwissTargetPrediction and BindingDB databases, *A. laxiflora* bioactive targets were accomplished [53,54]. The databases were queried with selected bioactive molecule canonical SMILES and 2D-SDF retrieved from PubChem. Only potential targets with probability scores of >0.70 and similarity scores of >0.70 were selected from the SwissTargetPrediction and BindingDB databases, respectively, with limitations to ‘*Homo sapiens’* as species. Screened potential targets were compiled in an Excel file and verified for duplicates. All the de-duplicated targets were standardized and unified using UniProtKB databases and saved as potential targets for further analysis.

### 4.3. Collection of Potential HCC-Related Protein Targets

HCC-associated protein targets were probed and collected from three public databases of DisGeNET [https://www.disgenet.org/search (accessed on 24 September 2023)], GeneCards [https://www.genecards.org/ (accessed on 24 September 2023)], and Comparative Toxicogenomics Database (CTD), [https://ctdbase.org/ (accessed on 24 September 2023)] [55,56,57]. Keywords for searching the databases were liver carcinoma, hepatocellular carcinoma, hepatic cancer, hepatic carcinoma, and hepatoma.

### 4.4. Potential Anti-HCC Targets Retrieval

The intersection targets between the screened targets of bioactive compounds and HCC-related protein targets were identified using the Venny 2.1.0 [58]. Retrieved overlapping targets were recognized as key anti-HCC targets.

### 4.5. Bioactive-Target Network Construction

By employing Cytoscape v3.9.1 [https://cytoscape.org/ (accessed on 26 September 2023)], a bioactive-target (BA-TAR) network was built to examine the multi-component interaction between bioactives and potential anti-HCC targets [59]. The constructed network was analyzed for topological characteristics using the cytoNCA plugin with the “degree value” setting [48]. Bioactive compounds with high degree value were identified as the core compounds for treating HCC.

### 4.6. Protein–Protein Interactions (PPI) Network Construction and Hub Gene Identification

The intersecting anti-HCC targets were queried in the STRING v11.5 [https://string-db.org/ (accessed on 26 September 2023)] database at a 0.7 confidence score and the “*Homo sapiens*” species limitation to find probable inter-target relationships [60]. The string analysis results were then imported to Cytoscape v3.9.1 to construct a visual PPI network for topological analysis. The topological parameters such as betweenness centrality value, degree value, and closeness centrality value were used to assess the node’s importance in the constructed network. These values were used to identify hub genes by setting cutoff criteria as the values higher than the median value. Furthermore, the MCODE plugin was used to perform cluster analysis of the PPI network.

### 4.7. Gene Ontology (GO) and KEGG Pathway Enrichment Analyses

To understand how *A. laxiflora* bioactives can be used to treat HCC, we analyzed potential anti-HCC core targets using the DAVID 2021 functional annotation tool [https://david.ncifcrf.gov/tools.jsp (accessed on 7 October 2023)] [61]. The analysis of GO and KEGG pathway enrichment was determined using the intersecting protein targets by entering their official gene symbols with *Homo sapiens* as the selected species. We applied filters to gene count from largest to smallest (*p*-value < 0.01, and FDR < 0.01) to sort the data and obtained the top 10 GO biological processes (BP), cellular component (CC) terms, molecular functions (MF), and the top 20 KEGG pathways [62]. Finally, SRPlot [https://www.bioinformatics.com.cn/en (accessed on 29 April 2024)] was used to prepare bubble and sanky plots for the GO terms and KEGG pathways, respectively.

### 4.8. Bioactive-Target-Pathway Network Construction

A bioactive-target-pathway network was established using Cytoscape v3.9.1 to describe the treatment mechanism of *A. laxiflora* for HCC. The network’s nodes with varied colors and forms represent key bioactives, hub gene targets, or disease pathways, while the edges connecting them show the links.

### 4.9. Molecular Docking

The molecular docking of pharmacologically promising phytoconstituents of *A. laxiflora* was conducted against the protein targets sorted out from the protein–protein interaction (PPI). The network pharmacology elucidated the crystal structures of the top eight hub genes, all of which were acquired from the protein data bank (PDB) in 3D format [63,64]. The following criteria were employed in selecting 3D structures of the suitable target proteins: (i) X-ray crystal structures exhibiting better resolution; (ii) if many structures were accessible for the same protein, then priority was given to the structures with the best resolution; (iii) co-crystallized proteins with ligands; (iv) the proteins extracted from humans were desired.

The 2D structures of phytoconstituents were obtained from the PubChem database [https://pubchem.ncbi.nlm.nih.gov/ (accessed on 10 February 2023)] and transformed into 3D chemical structures by the Chem3D 20.0 software. The PyMOL 2.4.0 software was employed to prepare the protein for docking, which involved the deletion of non-protein and/or water molecules, extracting the bound ligand from the protein complex, the addition of polar hydrogens, and repairing the missing atoms of amino acids [65]. AutoDock 4.2.1. and AutoDock Vina 1.1.2 were employed for the molecular docking [66,67]. For the docking simulations, a suitable grid box (20 Å) was created for each protein [AKT1 (PDB: 3O96), STAT3 (PDB: 6NUQ), MMP9 (PDB: 1GKC), EGFR (PDB: 2RGP), ESR1 (PDB: 1GWQ), MAPK1 (PDB: 1TVO), PTGS2 (PDB: 5F19), and SRC (PDB: 2BDF)] around the bound ligands, accordingly. The phytoligands considered for docking were Quercetin (PubChem ID: 5280343), 3-Acetylursolic acid (PubChem ID: 6475119), and 3-Acetyloleanolic acid (PubChem ID: 151202). The docking results were examined using BIOVIA Discovery Studio Visualizer [68].

### 4.10. Molecular Dynamics (MD) Simulations

The top-three high scoring complexes obtained from the molecular docking study were subjected to MD simulations using the Desmond module of the Schrodinger to further understand the stability of the protein–ligand (PL) complex [69,70]. Each PL complex was subjected to the system builder module where the PL complex was solvated with the TIP3P water model within the periodic boundary conditions, using an orthorhombic-shaped simulation box [71]. To manage the electroneutrality, Na^+^ or Cl^−^ ions were added. The NPT ensemble available within the Desmond package was used for minimization and relaxation [70]. The OPLS4 force field was used during all simulations [72] for a total of 100 ns with an interval of 100 ps. The temperature (300 K) and pressure (1.01325 bar) were maintained by the Nose–Hoover thermostat and Martyna–Tobias–Klein barostat with isotropic coupling [73,74,75]. Data analysis like root-mean-square deviations (RMSD) and protein–ligand interactions were analyzed using the simulation interaction diagram (SID) panel available in Schrödinger.

### 4.11. Correlation Analysis of Hub Gene Expression and HCC Patient Prognosis

The Kaplan–Meier mapping tool [http://kmplot.com/analysis/ (accessed on 22 October 2023)] was hired to evaluate the prognostic value of hub target genes. This tool provides information on gene expression data and the survival of liver cancer. The samples of liver cancer patients were categorized into different groups based on varied expression levels to analyze overall survival (OS) and relapse-free survival (RFS). Kaplan–Meier survival analysis was plotted using 95% confidence intervals (CI) and hazard ratios (HR) to investigate the hub target genes.

## 5. Conclusions

Given the complex nature of natural product metabolomes and the high cost of *in-house* screening for their roles and mechanisms in various diseases, network pharmacology-based analysis is considered a valuable and convenient approach to facilitate the task, more effectively. The current network pharmacology-based analysis of *A. laxiflora* suggested that compounds (quercetin, 3-acetylursolic acid, and 3-acetyloleanolic acid) in this plant can influence key cancer-associated targets EGFR, AKT1, SRC, and MAPK1 through the PI3K-Akt, MAPK, Ras, and Rap1 signaling pathways. These targets are implicated in cancer cell proliferation, angiogenesis, invasion, metastasis, and drug resistance. Further, to identify the precise target and to eliminate the off-targets, we performed molecular docking and MD analysis. Molecular docking and dynamics analyses allowed us to authenticate the findings and reasoned that 3-acetylursolic acid and quercetin have a strong binding affinity towards AKT1. Besides the ligands, the identified target AKT1 and MAPK are overexpressed in numerous cancer isoforms, along with sharing crosstalk in the cancer signaling pathways. Hence, our study opens avenues for cancer biologists to unravel the mechanistic insights into potential liver cancer/HCC treatments. Despite interesting research outcomes, our study has few limitations. This HCC involves complex pathological events, and the success rate of chemotherapeutic agents varies in each stage, significantly. This may also imply HCC progression stages while selecting an appropriate treatment approach. Further, different doses of phytoconstituents *A. laxiflora* against HCC should be considered and further experimental data are warranted to validate our results. In the future, we intend to explore the mechanism of action of phytoconstituents of *A. laxiflora* by conducting suitable in vitro experiments.

## Figures and Tables

**Table 1:** General information on screened bioactives of *A. laxiflora*.

PubChem ID	Bioactive	SMILES	BA	DL
5280343	Quercetin	C1=CC(=C(C=C1C2=C(C(=O)C3=C(C=C(C=C3O2)O)O)O)O)O	0.55	0.52
62453	4-Vinylphenol	C=CC1=CC=C(C=C1)O	0.55	0.29
2999413	Zeranol	C[C@H]1CCC[C@@H](CCCCCC2=C(C(=CC(=C2)O)O)C(=O)O1)O	0.55	0.5
151202	3-Acetyloleanolic acid	CC(=O)O[C@H]1CC[C@]2([C@H](C1(C)C)CC[C@@]3([C@@H]2CC=C4[C@]3(CC[C@@]5([C@H]4CC(CC5)(C)C)C(=O)O)C)C)C	0.85	0.57
6475119	3-Acetoxyursolic acid	C[C@@H]1CC[C@@]2(CC[C@@]3(C(=CC[C@H]4[C@]3(CC[C@@H]5[C@@]4(CC[C@@H](C5(C)C)OC(=O)C)C)C)[C@@H]2[C@H]1C)C)C(=O)O	0.85	0.84
91477	Cholest-4-en-3-one	C[C@H](CCCC(C)C)[C@H]1CC[C@@H]2[C@@]1(CC[C@H]3[C@H]2CCC4=CC(=O)CC[C@]34C)C	0.55	0.62
5742590	β-Sitosterol-3-O-β-D-glucopyranoside	CC[C@H](CC[C@@H](C)[C@H]1CC[C@@H]2[C@@]1(CC[C@H]3[C@H]2CC=C4[C@@]3(CC[C@@H](C4)O[C@H]5[C@@H]([C@H]([C@@H]([C@H](O5)CO)O)O)O)C)C)C(C)C	0.55	0.5
10140	Glycocholic acid	C[C@H](CCC(=O)NCC(=O)O)[C@H]1CC[C@@H]2[C@@]1([C@H](C[C@H]3[C@H]2[C@@H](C[C@H]4[C@@]3(CC[C@H](C4)O)C)O)O)C	0.56	0.29
6452096	Ethyl iso-allocholate	CCOC(=O)CC[C@@H](C)[C@H]1CC[C@@H]2[C@@]1([C@H](C[C@H]3[C@H]2[C@@H](C[C@H]4[C@@]3(CC[C@H](C4)O)C)O)O)C	0.55	0.39
538589	2*H*-Pyran-2-one, tetrahydro-4-hydroxy-6-pentyl-	CCCCCC1CC(CC(=O)O1)O	0.55	0.29
620007	4-Fluoro-2-nitroaniline, 5-[4-(pyrrolidin-1-yl)carbonylmethylpiperazin1-yl]-	C1CCN(C1)C(=O)CN2CCN(CC2)C3=C(C=C(C(=C3)N)[N+](=O)[O-])F	0.55	0.46
253193	Phaeophorbide A	CCC1=C(C2=NC1=CC3=C(C4=C([C@@H](C(=C5[C@H]([C@@H](C(=CC6=NC(=C2)C(=C6C)C=C)N5)C)CCC(=O)O)C4=N3)C(=O)OC)O)C)C	0.56	0.6
14135395	Byzantionoside B	CC1=CC(=O)CC([C@H]1CC[C@@H](C)O[C@H]2[C@@H]([C@H]([C@@H]([C@H](O2)CO)O)O)O)(C)C	0.55	0.6
551497	D-galactitol, 3,6-anhydro-1,2,4,5-tetra-o-methyl-	COCC(C1C(C(CO1)OC)OC)OC	0.55	0.29

**Table 2:** The degree analysis of screened bioactive compounds.

PubChem ID	Bioactive Compound	Degree
5280343	Quercetin	94
6475119	3-Acetoxyursolic acid	35
151202	3-Acetyloleanolic acid	34
2999413	Zeranol	24
91477	Cholest-4-en-3-one	23
62453	4-Vinylphenol	12
6452096	Ethyl iso-allocholate	8
10140	Glycocholic acid	4
538589	2*H*-Pyran-2-one, tetrahydro-4-hydroxy-6-pentyl-	2
5742590	β-Sitosterol-3-O-β-D-glucopyranoside	2
620007	4-Fluoro-2-nitroaniline, 5-[4-(pyrrolidin-1-yl)carbonylmethylpiperazin1-yl]-	1
551497	D-galactitol, 3,6-anhydro-1,2,4,5-tetra-o-methyl-	0

**Table 3:** The general description of the PPI network hub genes.

Hub Genes	Degree	Betweenness Centrality	Closeness Centrality
EGFR	38	2456.533	0.513514
SRC	37	1730.981	0.485401
STAT3	35	1978.653	0.496269
HSP90AB1	28	1993.469	0.475
AKT1	25	1173.136	0.5
ESR1	25	2190.166	0.503788
PTGS2	19	1503.855	0.457045
MAPK1	19	1499.365	0.434641
ALB	19	1962.353	0.471631
TLR4	18	1014.664	0.455479
MMP9	18	651.2776	0.446309
CYP1A1	18	725.4012	0.413043
CYP19A1	16	848.6445	0.410494
AR	15	760.8222	0.438944
NR3C1	13	407.2651	0.415625

**Table 4:** Top 10 highly enriched KEGG pathways.

Pathway ID	Pathway Name	*p*-Value	Gene Count	Hub Gene Count	Enriched Gene IDs
hsa01100	Metabolic pathways	0.005864	38	3	MAOB, PLA2G1B, MAOA, GLO1, AKR1B1, ALOX12, PYGL, HMGCR, CYP3A4, PTGS2, CYP19A1, PIK3CG, PTGS1, CYP17A1, HSD11B1, HSD11B2, CA3, CA2, HSD17B1, ALOX5, HSD17B2, CA9, XDH, ACP1, CA12, CBR1, SRD5A2, AKR1C1, PLA2G2A, AKR1C3, AKR1A1, DHCR24, AKR1B10, TST, CYP1A2, CYP1A1, UGT2B7, PTGES
hsa05200	Pathways in cancer	1.46 × 10^−12^	35	8	ALK, GSK3B, HSP90AB1, FLT3, FLT4, PIK3R1, PTGS2, RELA, EGFR, IGF1R, TERT, PIM1, AKT1, MAPK1, PDGFRB, PDGFRA, MAP2K1, MAP2K2, DAPK1, MMP2, STAT3, PRKCA, F2, MMP9, ESR1, PGF, PTK2, ESR2, VEGFA, MAPK10, AR, CDK6, KIT, CDK2, MET
hsa04151	PI3K-Akt signaling pathway	4.93 × 10^−11^	27	5	GSK3B, FLT1, HSP90AB1, FLT3, FLT4, PIK3R1, RELA, EGFR, PIK3CG, IGF1R, KDR, AKT1, MAPK1, PDGFRB, PDGFRA, NTRK2, MAP2K1, MAP2K2, PRKCA, PGF, PTK2, VEGFA, CDK6, KIT, CDK2, MET, TLR4
hsa05208	Chemical carcinogenesis-reactive oxygen species	8.13 × 10^−14^	25	5	SRC, AHR, PIK3R1, RELA, EGFR, CYP1B1, AKT1, MAPK1, MAP2K7, ACP1, PTPN1, CBR1, MAP2K1, MAP2K2, AKR1C1, AKR1C3, AKR1A1, AKR1C2, PTK2, VEGFA, MAPK10, CYP1A2, CYP1A1, NOX4, MET
hsa04010	MAPK signaling pathway	5.90 × 10^−11^	25	3	FLT1, FLT3, FLT4, RELA, EGFR, IGF1R, MKNK1, KDR, AKT1, MAPK1, MAP2K7, MAP2K6, PDGFRB, PDGFRA, NTRK2, MAP2K1, MAP2K2, MAP3K1, PRKCA, PGF, CDC25B, VEGFA, MAPK10, KIT, MET
hsa05207	Chemical carcinogenesis-receptor activation	2.39 × 10^−13^	24	9	MAP2K1, MAP2K2, HSP90AB1, SRC, VDR, STAT3, PRKCA, AHR, PIK3R1, CYP3A4, ESR1, EGFR, RELA, ESR2, VEGFA, AR, CYP1A2, CYP1A1, CYP1B1, AKT1, MAPK1, PGR, PPARA, UGT2B7
hsa04014	Ras signaling pathway	1.81 × 10^−11^	23	3	PDGFRB, PDGFRA, NTRK2, MAP2K1, MAP2K2, FLT1, PLA2G1B, FLT3, FLT4, PLA2G2A, PRKCA, PIK3R1, EGFR, PGF, RELA, IGF1R, VEGFA, MAPK10, KIT, KDR, AKT1, MAPK1, MET
hsa04510	Focal adhesion	3.29 × 10^−9^	19	4	PDGFRB, PDGFRA, GSK3B, MAP2K1, FLT1, SRC, FLT4, PRKCA, PIK3R1, EGFR, PGF, PTK2, IGF1R, VEGFA, MAPK10, KDR, AKT1, MAPK1, MET
hsa04015	Rap1 signaling pathway	5.66 × 10^−9^	19	4	PDGFRB, PDGFRA, MAP2K1, MAP2K2, FLT1, SRC, FLT4, PRKCA, PIK3R1, EGFR, PGF, IGF1R, VEGFA, KIT, KDR, AKT1, MAPK1, MET, MAP2K6
hsa05206	MicroRNAs in cancer	2.07 × 10^−6^	19	5	PDGFRB, PDGFRA, MAP2K1, ABCC1, MAP2K2, ABCB1, STAT3, PRKCA, PIK3R1, PTGS2, MMP9, EGFR, CDC25B, VEGFA, CDK6, PIM1, CYP1B1, MAPK1, MET

**Table 5:** The binding energy (kcal/mol) of each ligand against the selected targets.

Ligand (PubChem ID)	MAPK1	AKT1	EGFR	SRC	STAT3	MMP9	PTGS2	ESR1
Quercetin (5280343)	−8.2 *	−9.8 *	−7.8 *	−7.8 *	−5.9 *	−8.8	−8.9	−8.0
3-Acetylursolic acid (6475119)	−9.2 *	−11.5 *	−8.9 *	−9.2 *	−6.9 *	−7.1	−4.6	−5.9
3-Acetyloleanolic acid (151202)	−9.4 *	−8.5 *	−8.2 *	−9.1 *	−6.8 *	−7.3	−3.6	−5.6

* The binding energies of these complexes are reported in our earlier work.

## Data Availability

Data are contained within the article.
